# Effects of Cognitive Nursing Combined with Continuous Nursing on Postpartum Mental State and Rehabilitation

**DOI:** 10.1155/2021/4131917

**Published:** 2021-12-02

**Authors:** Caijuan Liu, Dailing Xiao, Deping Han, Shimin Li, Tianli Zhu, Wenjuan Wang, Li Zhou, Wen Yan, Weiming Lu

**Affiliations:** ^1^Chengyang People's Hospital, Qingdao, China; ^2^Department of Operation room, Jinan Maternal & Children Health Care Hospital, Jinan, Shangdong, China; ^3^Department of Obstetric ward 2, Xinhua Hospital Affiliated to Medical College of Shanghai Jiaotong University, Shanghai 200092, China; ^4^Department of Obstetrics ward 1, Xinhua Hospital Affiliated to Medical College of Shanghai Jiaotong University, Shanghai 200092, China

## Abstract

**Purpose:**

This study is aimed at exploring the effects of cognitive nursing combined with continuous nursing on postpartum mental state and rehabilitation.

**Methods:**

Totally, 124 puerperas admitted to our hospital from January 2019 to January 2020 were selected and divided into a research group and a control group according to different nursing methods, with 62 cases in each group. The control group received routine care, while the research group received cognitive nursing combined with continuous nursing on this basis. The mental state, rehabilitation indicators, quality of life, incidence of complications, and nursing satisfaction were compared between the two groups after intervention.

**Results:**

Before nursing, there was no statistically significant difference in the SAS and SDS scores between the two groups (*P* > 0.05); after intervention, the SAS and SDS scores of the two groups were significantly reduced, and those of the research group were lower than those of the control group (*P* < 0.05). After intervention, the time of the first breastfeeding, duration of lochia rubra, length of hospital stay, and score of uterine contraction pain of the research group were lower than those of the control group (*P* < 0.05); the psychological function, physical function, material life, and social function scores of the research group were higher than those of the control group (*P* < 0.05); the incidence of complications in the research group was 4.84%, lower than 20.97% in the control group (*P* < 0.05); the nursing satisfaction of the research group was 96.77%, which was significantly higher than 83.87% in the control group (*P* < 0.05).

**Conclusions:**

Cognitive nursing combined with continuous nursing can effectively improve the mental state, shorten the length of hospital stay, increase the perceived well-being, and promote the physical rehabilitation in puerperas, which is worth promoting in clinical practice.

## 1. Introduction

A puerpera refers to a woman in the puerperium and parturition period. This is a necessary stage in the life activities of most puerperas and is often accompanied by a series of obvious psychological and physical stress reactions [1]. Due to the difficulty in adapting to the mother's role, complex emotions, and excessive worries, some puerperas have psychological problems such as anxiety and depression. Coupled with abdominal weight and fetal movement, these problems often produce negative emotional experiences, elevate the heart rate and blood pressure of puerperas, lead to sleep disorders and other adverse events, increase the risks of uterine inertia and postpartum contractions, and compromise the delivery process and the postpartum recovery of puerperas [2, 3]. Conventional obstetric nursing interventions in clinical practice often ignore the mental state and needs of puerperas and cannot ameliorate their negative state completely [4]. Therefore, we have been exploring a more effective postpartum nursing intervention for clinical practice. In recent years, as the living standards are improved, people have higher requirements for the quality of nursing services, and more humane, refined, and higher-quality nursing interventions are needed for puerperas in clinical practice. Cognitive nursing enables patients to have correct cognitive behaviors by correcting wrong cognition and ultimately alleviates the condition and treats the disease [5]. Continuous nursing is the continuation of hospital care. It is a people-oriented, scientific, and systematic model that can provide professional guidance on infant care and health care for a certain time after discharge from the hospital and effectively improve the physical and mental health as well as the quality of life of puerperas [6]. There are few studies on cognitive nursing combined with continuous nursing. Hence, in this study, a total of 124 puerperas admitted to our hospital from January 2019 to January 2020 were selected to explore the effects of cognitive nursing combined with continuous nursing on postpartum mental state and rehabilitation of puerperas.

## 2. Materials and Methods

### 2.1. Subjects

Totally, 124 puerperas admitted to our hospital from January 2019 to January 2020 were selected as the subjects. The puerperas were divided into research group and control group, with 62 cases in each group. Randomisation was stratified by center with minimisation for age, gestational age, education level, and gravidity. The control group: 22-36 years old, with an average of (29.73 ± 4.28) years; the gestational age was 37-40 weeks, with an average of (39.25 ± 1.52) weeks; 33 postpartum women and 29 primiparous women; 14 cases with junior high school education, 19 with senior high school education, and 29 with university education. The research group: 21-37 years old, with an average of (29.80 ± 4.30) years; the gestational age was 36-40 weeks, with an average of (39.05 ± 1.43) weeks; 33 postpartum women and 29 primiparous women; 15 cases with junior high school education, 21 cases with senior high school education, and 26 cases with university education. There was no statistically significant difference in the general data between the two groups (*P* > 0.05). This study was approved by the appropriate ethics committee of our hospital.

Inclusion criteria: [1] puerperas who gave singleton birth naturally; [2] complete clinical data; [3] patients' informed consent. Exclusion criteria: [1] severe complications in childbirth; [2] severe heart, liver, kidney, and other organ diseases; [3] mental and cognitive-communication deficits.

### 2.2. Methods

The control group received routine nursing care as follows. The self-made postpartum care manual of our hospital was distributed and explained to the puerperas, and psychological counseling was provided to them. They and their families were informed of the hotline of the obstetrics department so that they could consult the department at any time. Their contact information was recorded for follow-up.

The research group underwent cognitive nursing combined with continuous nursing on the basis of the control group as follows. [1] A joint cognitive and continuous nursing team was established, with the head nurse as the team leader. Comprehensively understood the basic information of patients and families, communicate with patients, and analyze the psychological dynamics of patients. The root causes of patients with depression were evaluated. [2] Cognitive nursing care. The nurses communicated with the puerperas in time, encouraged them to tell their true emotions, listened to them carefully, analyzed their problems, corrected their distorted cognition, reshaped their cognition, and resolved their negative emotions such as depression and anxiety in an “empathetic” way. The family members were educated to increase their attention to the puerperas with care and encouragement to improve their self-confidence and cooperation. [3] A reasonable and scientific diet plan was developed according the health status of the puerperas, which included high-calorie, high-protein, high-vitamin, and easily digestible foods. [4] Continuous nursing care. A postdischarge nursing plan was formulated before the puerperas were discharged from the hospital, and a health record was established for them, for example, a return visit file to clarify the method, time, and content of return visits and record their relevant information. [5] The medical and nursing staff carried out a telephone follow-up within 1 week after the puerperas were discharged from the hospital to understand their current situation and provide health education and guidance on their life. The medical and nursing staff advised the family members to take care of the puerperas and take the initiative to take care of the newborns, so that the puerperas could face life with a positive attitude and avoid emotional fluctuations. [6] The medical and nursing staff could provide psychological counseling to the puerperas through text messages, phone calls, WeChat, etc., so that the puerperas could pour out their emotions. Meanwhile, the medical and nursing staff developed an exercise plan including respiratory training, postpartum yoga training for the research group to promote their recovery. [7] The medical and nursing staff conducted a home follow-up visit once a month to understand their family support and evaluate their mental and physical recovery and provided guidance for their future recovery.

### 2.3. Observation Indicators

#### 2.3.1. Mental State

After one-month intervention, the mental state was evaluated. The Self-Anxiety Scale (SAS) and Self-Depression Scale (SDS) were used to evaluate the mental state of the puerperas. There were 40 items in SAS and SDS, each with an average of 20 items, and the score of each item was 1-4. SAS rating standards [7]: (1) ≥70 scores = severe anxiety; (2) 60 − 69 scores = moderate anxiety; (3) 50 − 59 scores = mild anxiety; and (4) <50 scores = normal. The higher the score, the more serious the anxiety. SDS rating standards [8]: (1) <50 scores = normal; (2) 50 − 59 = mild depression; (3) 60 − 69 = moderate depression; and (4) ≥70 = severe depression. The higher the score, the more severe the depression.

#### 2.3.2. Postpartum Rehabilitation Indicators

Postpartum rehabilitation indicators including the time of the first breastfeeding, duration of lochia rubra, length of hospital stay, and score of uterine contraction pain were collected and compared. Among them, the uterine contraction pain score was measured by the McGill Pain Questionnaire (MPQ). The MPQ score was 0-5: 0 = no uterine contraction pain, 5 = severe uterine contraction pain, and the degree of pain was proportional to the score.

#### 2.3.3. Quality of Life

After one-month intervention, the quality of life was evaluated by the Generic Quality of Life Inventory-74 (GQOLI-74) [9], mainly divided into mental function, physical function, material life, and social function. The total score of GQOLI-74 was 100. The higher the score, the better the quality of life.

#### 2.3.4. Complications

After one-month intervention, the complications including asitia, insomnia, and mastitis were monitored. The complication rate was obtained. Comparison of the incidence of complications between the two groups after intervention.

#### 2.3.5. Nursing Satisfaction

The self-made satisfaction inventory of our hospital was used to evaluate the service attitude, communication skills, nursing skills, humane care, and health anonymously. The total score was 100. A score of >90 was considered satisfactory; a score of 70-90 was basically satisfactory; a score of <70 was unsatisfactory. The higher the score, the higher the satisfaction. Satisfaction = (satisfaction rate + relatively satisfactory rate)/total number of cases × 100%.

## 3. Results

### 3.1. Comparison of the Mental State of the Two Groups before and after Intervention

**Figure 1:**
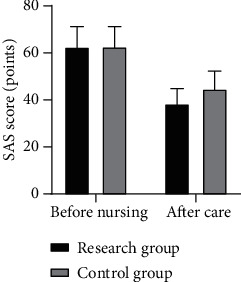
SAS scores of the two groups before and after intervention.

**Figure 2:**
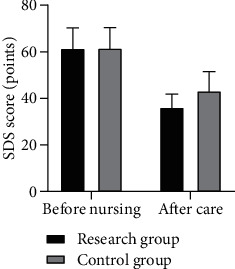
SDS scores of the two groups before and after intervention.

Before nursing, there was no statistically significant difference in SAS and SDS scores between the two groups (*P* > 0.05). After intervention, the SAS and SDS scores of the two groups were significantly reduced, and those of the research group were lower than those of the control group (*P* < 0.05); see Table 1, Figures 1 and 2.

### 3.2. Comparison of Postpartum Rehabilitation Indicators between the Two Groups

After intervention, the time of the first breastfeeding, duration of lochia rubra, length of hospital stay, and score of uterine contraction pain of the research group were lower than those of the control group (*P* < 0.05); see Table 2.

### 3.3. Comparison of the Quality of Life Scores between the Two Groups after Intervention

After intervention, the psychological function, physical function, material life, and social function scores of the research group were higher than those of the control group (*P* < 0.05); see Table 3.

### 3.4. Comparison of the Incidence of Complications between the Two Groups after Intervention

The incidence of complications of the research group was 4.84%, lower than 20.97% of the control group (*P* < 0.05); see Table 4.

### 3.5. Comparison of Nursing Satisfaction between the Two Groups of Puerperas

The nursing satisfaction rate of the research group was 96.77%, which was significantly higher than 83.87% of the control group (*P* < 0.05); see Table 5.

## 4. Discussion

During pregnancy, most women are prone to life stress, hormone secretion, physical discomfort and excessive worries, and negative emotions such as depression and anxiety after delivery, thus, compromising the postpartum recovery of the puerperas and the relationship between them and their family [10]. According to research reports [11, 12], 3.5% to 33% of puerperas in other countries and 4.5% to 20% in China have postpartum depression, which seriously threatens the health and safety of puerperas and babies. Due to lack of labor experience and knowledge, primiparas often have various worries during pregnancy, and due to the change of roles after delivery, some puerperas experience obvious loneliness [13]. Therefore, it is necessary to carry out nursing interventions for puerperas to relieve their negative emotions and promote their postpartum psychological and physical recovery.

In recent years, with the transformation of the nursing model, the concept of nursing care has undergone a major change from “disease-centered” to “people-centered,” which gives play to the central role of puerperas in postpartum rehabilitation [14]. It scientifically extends the nursing service for puerperas from the hospital to the community and the family and does not affect the coordination and continuity between the nursing inside and outside the hospital, ensuring the continuity of nursing services for puerperas after leaving the hospital [15]. Continuous services are provided to puerperas after discharge, mainly by phone calls, text messages, and follow-ups. Health education on the healthcare knowledge of themselves and their newborns and psychological interventions are provided to puerperas, so as to make them aware of the harm of their own emotional changes and inform them of postpartum self-care and newborn care in time to reduce postpartum infections [16]. In addition, from the perspective of psychological defense mechanism, the doubts of the puerperas can be resolved by telephone at any time, which improves the adaptability during the puerperium, enhances the cognition of the puerperium healthcare and the nursing of the newborn, and prevents the occurrence of bad emotions caused by various factors. Meanwhile, text messages, WeChat, phone calls, and follow-up provide a window for the puerperas to release their psychological pressure and reduce their psychological burden. Relevant clinical studies have shown [17] that effective continuous nursing care for the puerperas after delivery can increase their knowledge of pelvic floor dysfunction, thereby arousing their enthusiasm and initiative. Cognitive nursing is a more effective treatment method, which can effectively alleviate negative mental states and reduce anxiety and depression and other negative behaviors and cognition [18]. Relevant articles have shown [6] that cognitive behavior can correct the negative thinking of puerperas, replace negative avoidance, change negative cognition, and effectively ameliorate anxiety and depression and other adverse symptoms. Hinkle et al. [19] has found that negative emotions during the puerperium may enhance the body's stress ability, and effective nursing interventions can reduce the negative emotions after delivery and the postpartum complications, and improve the mental state of puerperas. This study mainly explores the effect of cognitive nursing combined with continuous nursing on the postpartum mental state and rehabilitation of puerperas, and the nursing effect is definite. The results of this study showed that after intervention, the SAS and SDS scores of the two groups were significantly reduced, and those of the research group were lower than those of the control group, indicating that cognitive nursing combined with continuous nursing can significantly relieve postpartum depression. After intervention, the time of the first breastfeeding, duration of lochia rubra, length of hospital stay, and score of uterine contraction pain of the research group were lower than those of the control group, indicating that cognitive nursing combined with continuous nursing can speed up the physical recovery of puerperas. After intervention, the scores of psychological function, physical function, material life, and social function of the research group were higher than those of the control group, showing that cognitive nursing combined with continuous nursing can effectively improve the quality of life of puerperas. The complication rate of the research group was 4.84%, lower than 20.97% of the control group, and the nursing satisfaction rate of the research group was 96.77%, significantly higher than 83.87% of the control group, indicating that this nursing model can not only reduce the incidence of postpartum complications but also improve patient satisfaction.

In conclusion, cognitive nursing combined with continuous nursing can effectively improve the mental state of puerperas, shorten the length of hospital stay, enhance the health awareness, and promote the physical rehabilitation. It is worth promoting in clinical practice.

## Figures and Tables

**Table 1:** SAS and SDS scores of the two groups before and after intervention (^−^*χ* ± s).

Group	SAS scores		SDS scores	
	Before nursing	After intervention	Before nursing	After intervention
Research group (*n* = 62)	62.63 ± 9.15	38.44 ± 6.85^a^	61.58 ± 9.22	36.24 ± 6.05^a^
Control group (*n* = 62)	62.68 ± 9.11	44.68 ± 8.07^a^	61.62 ± 9.30	43.29 ± 8.68^a^
*t*	0.030	4.642	0.024	5.247
*P*	0.976	<0.001	0.980	<0.001

Note: a represented that compared with that before nursing, *P* < 0.05.

**Table 2:** Comparison of the postpartum physiological state between the two groups (^−^*χ* ± s).

Group	Time of the first breastfeeding	Duration of lochia rubra (h)	Length of hospital stay (h)	Score of uterine contraction pain
Research group (*n* = 62)	15.87 ± 2.15	2.00 ± 1.01	3.33 ± 1.17	2.90 ± 1.30
Control group (*n* = 62)	23.63 ± 2.84	3.63 ± 1.53	6.05 ± 1.25	4.63 ± 1.51
*t*	17.150	7.001	12.510	6.837
*P*	<0.001	<0.001	<0.001	<0.001

**Table 3:** Comparison of the quality of life scores between the two groups after intervention (^−^*χ* ± s).

Group	Psychological function	Physical function	Material life	Social function
Research group (*n* = 62)	57.87 ± 5.59	64.82 ± 6.33	70.25 ± 7.18	67.22 ± 6.16
Control group (*n* = 62)	53.25 ± 5.44	59.36 ± 6.06	63.11 ± 5.46	60.36 ± 5.77
*t*	4.664	4.906	6.233	6.400
*P*	<0.001	<0.001	<0.001	<0.001

**Table 4:** Comparison of the incidence of complications between the two groups (*n*, %).

Group	Asitia	Insomnia	Mastitis	Total incidence
Research group (*n* = 62)	1 (1.61)	2 (3.23)	0 (0.00)	3 (4.84)
Control group (*n* = 62)	5 (8.06)	6 (9.68)	3 (4.84)	13 (20.97)
*χ* ^2^				7.176
*P*				0.007

**Table 5:** Comparison of the satisfaction rate between the two groups (*n*, %).

Group	Satisfactory	Basically satisfactory	Unsatisfactory	Satisfaction rate
Research group (*n* = 62)	48 (77.42)	12 (19.35)	2 (3.23)	60 (96.77)
Control group (*n* = 62)	24 (38.71)	28 (45.16)	10 (16.13)	52 (83.87)
*χ* ^2^				5.905
*P*				0.015

## Data Availability

The data used to support the findings of this study are available from the corresponding author upon request.
